# Precision Enhancement of Wireless Localization System Using Passive DOA Multiple Sensor Network for Moving Target

**DOI:** 10.3390/s22197563

**Published:** 2022-10-06

**Authors:** Chien-Bang Chen, Tsu-Yu Lo, Je-Yao Chang, Shih-Ping Huang, Wei-Ting Tsai, Chong-Yi Liou, Shau-Gang Mao

**Affiliations:** Graduate Institute of Commutation Engineering, National Taiwan University, Taipei 106, Taiwan

**Keywords:** wireless localization system, angle of arrival, Kalman Filter

## Abstract

Determining the direction-of-arrival (DOA) of any signal of interest has long been of great interest to the wireless localization research community for military and civilian applications. To efficiently facilitate the deployment of DOA systems, the accuracy of wireless localization is critical. Hence, this paper proposes a novel method to improve the prediction result of a wireless DOA localization system. By considering the signal variation existing in the complex environment, the actual location of the target can be determined including the maximum prediction error. Moreover, the scenario of the moving target is further investigated by incorporating the adaptive Kalman Filter algorithm to obtain the prediction route of the flying drone based on the accuracy assessment method. This proposed adaptive Kalman Filter is a high-efficiency algorithm that can filter out the noise in the multipath area and optimize the predicted data in real-time. The simulation result agrees well with the measured data and thus validates the proposed DOA system with the adaptive Kalman Filter algorithm. The measured DOA of the fixed radiation source obtained by a single base station and the moving route of a flying drone from a two-base station localization system are presented and compared with the calculated results. Results show that the prediction error in an outdoor region of $500\times 500{\;m}^{2}$ is about 10–20 m, which demonstrate the usefulness of the proposed wireless DOA system deployment in practical applications.

## 1. Introduction

The rapid development of radio communications has resulted in the increasing importance of wireless localization, thus leading to the significant boosting requirements and the complexity of wireless sensor networks. The localization system constructed by wireless communication is adopted to determine the location information of the mobile target. The Global Position System (GPS) and the cellular base station wireless system obtain the positions of the moving targets via the signals transmitted by multiple nodes and received by mobile devices [1,2,3,4,5,6]. Moreover, in modern military applications, the command system requires the ability to locate enemy signal emission sources to achieve the correct and rapid response capabilities. This passive detection system, which does not transmit any electromagnetic energy, can covertly determine the location of the emission source, and has the advantages of radiation stealth, long distance and strong anti-interference ability. However, it is challenging to accurately localize a moving signal source in a complex environment by using the wireless sensor network due to the influence caused by multipath fading and non-line-of-sight (NLOS) transmission [7,8,9,10,11,12,13]. To overcome these critical issues in practical applications, the technology will be developed based on a sufficiently broad and essential foundation, including array signal processing, antennas and wave propagation, radio frequency (RF) circuit technology, data communications, software engineering, etc.

This paper explores the accurate angle and region of an actual target in the single-base station and two-base station localization systems experimentally and theoretically by including the measurement error of a direction-finding station. Those positions can be identified by determining the connection between the localization system’s prediction and the actual target’s location. Thus, the development of the multiple direction-finding base station system is established. Moreover, this paper utilizes the concept of the adaptive Kalman Filter algorithm to decrease the noise error and thus enhance the prediction accuracy of a moving target in the complex multipath wireless scenario.

To authors’ best knowledge, this paper first proposes a novel wireless localization system by using passive DOA multiple stations to theoretically predict and experimentally measure the moving target trace. The adaptive Kalman Filter combined with the probability distribution function of the target location is presented to verify the effectiveness and the precision of the presented wireless sensor network.

## 2. Related Works

The main application principle of the so-called radio direction finding and positioning technology is to determine the location of the target radiation source independently and passively through the propagation of electromagnetic waves. Direction-finding stations at different locations conduct direction-finding results based on the same radiation source and determine the direction of the measured target according to the difference in amplitude, phase and frequency induced by the incident electromagnetic wave in the direction-finding system. Thus, the areas of intersection among the multiple direction-finding stations address the localizations of moving targets. Generally, three basic positioning methods using the strength of the received signal (received-signal-strength-indicator, RSSI) [14,15,16,17,18], the time delay of the received signal (time-difference-of-arrival, TDOA) and the angle of the received signal (direction-of-arrival, DOA) were presented.

The RSSI method depends on the wave propagation channel attenuation model of the practical scenario. Hence, the distances between the sources and the receivers are estimated by measuring the RSSI, and the localization is carried out by multilateration with large errors [19,20,21,22]. The TDOA method provides reasonably high accuracy of localization and are independent of the propagation channel model. However, it requires critical time synchronization across the sensor nodes. Moreover, the number of sensor nodes should be larger than three and the error of target localization is increased outside the covered region of all sensor nodes [23,24,25,26,27,28]. The DOA based localization can be achieved by using only two sensor nodes with no need to synchronize each node. Without suffering from the drawbacks of the aforementioned methods, the DOA based localization may better accommodate some IoT applications because it is simple, robust and cost-effective and energy saving [29,30,31,32].

This paper proposes a wireless localization system using a passive DOA sensor network. The analysis of two-base station direction-finding location estimation is proposed in Section 3, including the angle variation of DOA technique. Section 4 discusses an adaptive algorithm based on the Kalman Filter that can precisely determine the localization of moving targets from the estimated signal angles. Section 5 illustrates the actual experimental results established by a localization system based on the methods proposed in Section 3 and Section 4. Section 6 draws a conclusion and proposes future work.

## 3. Multi-Stations Localization Techniques

### 3.1. System Description

**Figure 1:**
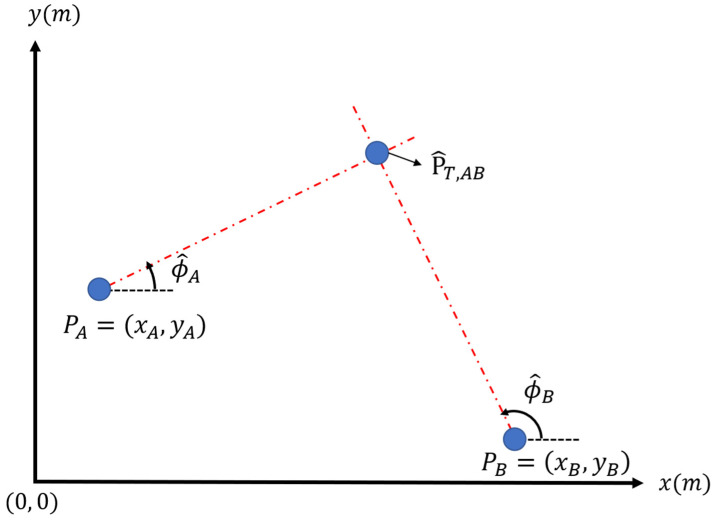
Two-base stations localization system.

The two-base station localization system is shown in Figure 1. Station A is located at $P_{A}=\left(x_{A},\;y_{A}\right)$ and Station B is located at $P_{B}=\left(x_{B},\;y_{B}\right)$. Since ${\hat{\phi }}_{A}$ and ${\hat{\phi }}_{B}$ are the estimated directions given by Station A and Station B, it is easy to predict the target’s location by drawing two dotted lines using two stations’ locations and predicted directions. The intersection of these dotted lines is the prediction point of the target ${\hat{P}}_{T}=\left({\hat{x}}_{T},\;{\hat{y}}_{T}\right)$.

To find out the possible region of the actual target point, we assumed that the direction estimation by Stations A and B have errors and the errors are $\Delta \phi_{A}$ and $\;\Delta \phi_{B}$, respectively. Thus, the actual direction of the target $\phi_{A}$ and $\phi_{B}$ should be reasonably constrained by:

(1) $${\hat{\phi }}_{A}-\Delta \phi_{A}\leq {\hat{\phi }}_{A}\leq {\hat{\phi }}_{A}+\Delta \phi_{A}$$

(2) $${\hat{\phi }}_{B}-\Delta \phi_{B}\leq {\hat{\phi }}_{B}\leq {\hat{\phi }}_{B}+\Delta \phi_{B}$$

Therefore, four solid lines, $L_{1}$, $L_{2}$, $L_{3}$ and $L_{4}$, are obtained with four different oblique angles, ${\hat{\phi }}_{A}-\Delta \phi_{A}$, ${\hat{\phi }}_{A}+\Delta \phi_{A}$, ${\hat{\phi }}_{B}-\Delta \phi_{B}$ and ${\hat{\phi }}_{B}+\Delta \phi_{B}$. Then, these four solid lines should bound to the actual target point. The equations of these four lines are presented below.

(3) $$y=\tan\left({\hat{\phi }}_{B}-\Delta \phi_{B}\right)\left(x-x_{B}\right)+y_{B}$$

(4) $$y=\tan\left({\hat{\phi }}_{A}+\Delta \phi_{A}\right)\left(x-x_{A}\right)+y_{A}$$

(5) $$y=\tan\left({\hat{\phi }}_{B}+\Delta \phi_{B}\right)\left(x-x_{B}\right)+y_{B}$$

(6) $$y=\tan\left({\hat{\phi }}_{A}-\Delta \phi_{A}\right)\left(x-x_{A}\right)+y_{A}$$

In addition, four coordinates of the intersection region are obtained, i.e., ${\hat{P}}_{1,AB}=\left({\hat{x}}_{1,\;AB},{\hat{y}}_{1,\;AB}\right)$, ${\hat{P}}_{2,AB}=\left({\hat{x}}_{2,\;AB},{\hat{y}}_{2,\;AB}\right)$, ${\hat{P}}_{3,AB}=\left({\hat{x}}_{3,\;AB},{\hat{y}}_{3,\;AB}\right)$, and ${\hat{P}}_{4,AB}=\left({\hat{x}}_{4,\;AB},{\hat{y}}_{4,\;AB}\right)$, in Equations (7)–(14):

(7) $${\hat{x}}_{1,AB}=\frac{y_{A}-y_{B}+\tan\left({\hat{\phi }}_{B}-\Delta \phi_{B}\right)x_{B}-\tan\left({\hat{\phi }}_{A}-\Delta \phi_{A}\right)x_{A}}{\tan\left({\hat{\phi }}_{B}-\Delta \phi_{B}\right)-\tan\left({\hat{\phi }}_{A}-\Delta \phi_{A}\right)}$$

(8) $${\hat{x}}_{2,AB}=\frac{y_{A}-y_{B}+\tan\left({\hat{\phi }}_{B}-\Delta \phi_{B}\right)x_{B}-\tan\left({\hat{\phi }}_{A}+\Delta \phi_{A}\right)x_{A}}{\tan\left({\hat{\phi }}_{B}-\Delta \phi_{B}\right)-\tan\left({\hat{\phi }}_{A}+\Delta \phi_{A}\right)}$$

(9) $${\hat{x}}_{3,AB}=\frac{y_{A}-y_{B}+\tan\left({\hat{\phi }}_{B}+\Delta \phi_{B}\right)x_{B}-\tan\left({\hat{\phi }}_{A}+\Delta \phi_{A}\right)x_{A}}{\tan\left({\hat{\phi }}_{B}+\Delta \phi_{B}\right)-\tan\left({\hat{\phi }}_{A}+\Delta \phi_{A}\right)}$$

(10) $${\hat{x}}_{4,AB}=\frac{y_{A}-y_{B}+\tan\left({\hat{\phi }}_{B}+\Delta \phi_{B}\right)x_{B}-\tan\left({\hat{\phi }}_{A}-\Delta \phi_{A}\right)x_{A}}{\tan\left({\hat{\phi }}_{B}+\Delta \phi_{B}\right)-\tan\left({\hat{\phi }}_{A}-\Delta \phi_{A}\right)}$$

(11) $${\hat{y}}_{1,AB}=\tan\left({\hat{\phi }}_{A}-\Delta \phi_{A}\right)\left({\hat{x}}_{1,AB}-x_{A}\right)+y_{A}$$

(12) $${\hat{y}}_{2,AB}=\tan\left({\hat{\phi }}_{A}+\Delta \phi_{A}\right)\left({\hat{x}}_{2,AB}-x_{A}\right)+y_{A}$$

(13) $${\hat{y}}_{3,AB}=\tan\left({\hat{\phi }}_{A}+\Delta \phi_{A}\right)\left({\hat{x}}_{3,AB}-x_{A}\right)+y_{A}$$

(14) $${\hat{y}}_{4,AB}=\tan\left({\hat{\phi }}_{A}-\Delta \phi_{A}\right)\left({\hat{x}}_{4,AB}-x_{A}\right)+y_{A}$$

**Figure 2:**
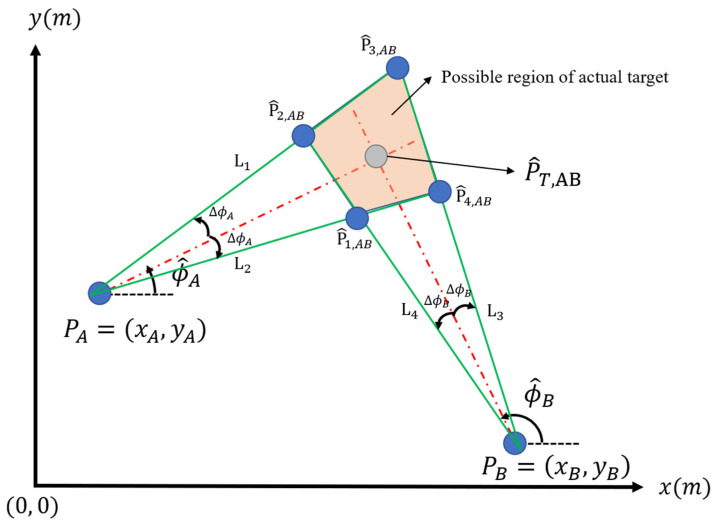
Possible region of the actual target.

Figure 2 shows that the actual location of the target should reasonably localize inside the quadrilateral ${\hat{P}}_{1,AB}{\hat{P}}_{2,AB}{\hat{P}}_{3,AB}{\hat{P}}_{4,AB}$. Moreover, the localization system’s accuracy is always evaluated by comparing the distance between the prediction point and the actual target. Thus, the error function at ${\hat{P}}_{T,AB}$ is the maximum distance between ${\hat{P}}_{T,AB}$ and each point inside the bounded region are obtained. Thus, this error function, called $\Lambda_{AB}\left({\hat{P}}_{T,AB}\right),\;$can be written as:

(15) $$\Lambda_{AB}=\max\left(\bar{{\hat{P}}_{T,\;AB}{\hat{P}}_{1,\;AB}},\bar{{\hat{P}}_{T,\;AB}{\hat{P}}_{2,\;AB}},\;\bar{{\hat{P}}_{T,\;AB}{\hat{P}}_{3,\;AB}},\;\bar{{\hat{P}}_{T,\;AB}{\hat{P}}_{4,\;AB}}\right)$$

where $\bar{ab}$ denotes the distance between a and b. From Equations (3)–(15), the estimation error is determined by the location of the prediction point and Stations A and B.

**Figure 3:**
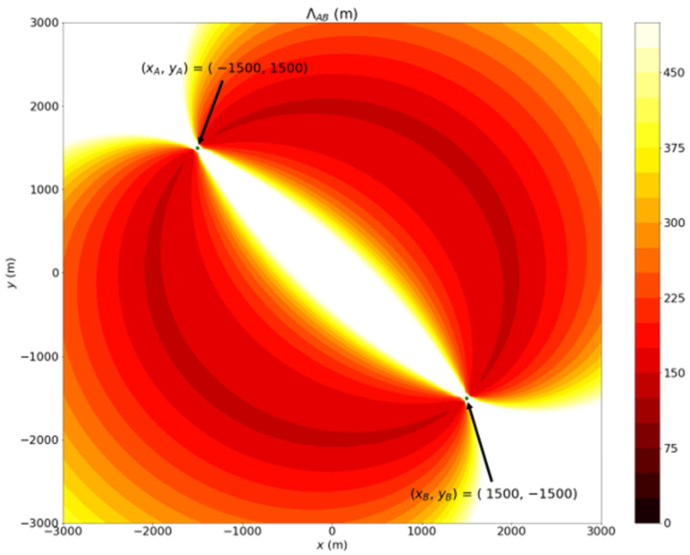
Example of the distribution of *Λ_AB_*.

Figure 3 shows the $\Lambda_{AB}$ for different locations of ${\hat{P}}_{T}$, $-3000\leq \;{\hat{x}}_{T}\leq 3000$ and $-3000\leq \;{\hat{y}}_{T}\leq 3000$, when $P_{A}=\left(x_{A},\;y_{A}\right)=\left(-1500,1500\right)$, $P_{B}=\left(x_{B},\;y_{B}\right)=\left(1500,-1500\right)$ and $\;\Delta \phi_{A}=\;\Delta \phi_{B}=2{}^{\circ}$. The contours in Figure 3 indicate that the values of $\Lambda_{AB}$ at different points on the plane are varied. In addition, the contours illustrate that the prediction’s accuracy of the two stations’ localization system will decrease if the target is close to the crossing-line of Station A and B or far away from Station A and B, as depicted in the white color region of Figure 3. Therefore, the two-base station localization system’s covered region would be restricted if the system’s precision is constrained.

### 3.2. Triple Base Stations Localization System

**Figure 4:**
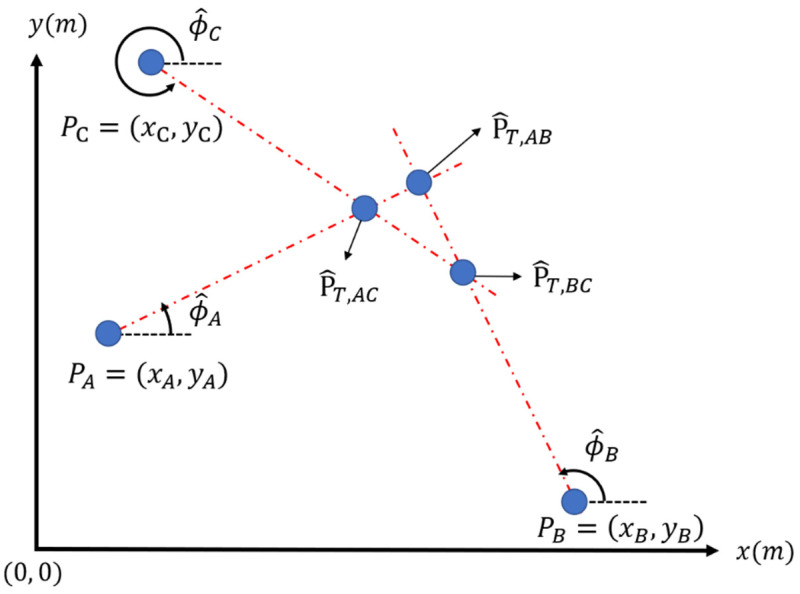
Triple base stations localization system.

To extend the result into a multiple-stations localization system, the system has another Station C located at ($x_{C}$, $y_{C}$) considered in this section. As depicted in Figure 4, three different prediction points of the target, ${\hat{P}}_{T,AB}$, ${\hat{P}}_{T,AC}$ and ${\hat{P}}_{T,BC}$, are obtained by using three different stations’ pairs, (A, B), (A, C), and (B, C). Since the direction predictions have errors, these three prediction points may not be at the same point. Therefore, we will analyze how to choose a proper target estimation with minimum estimation error using these three prediction points based on the error analysis in the two-base station system.

**Figure 5:**
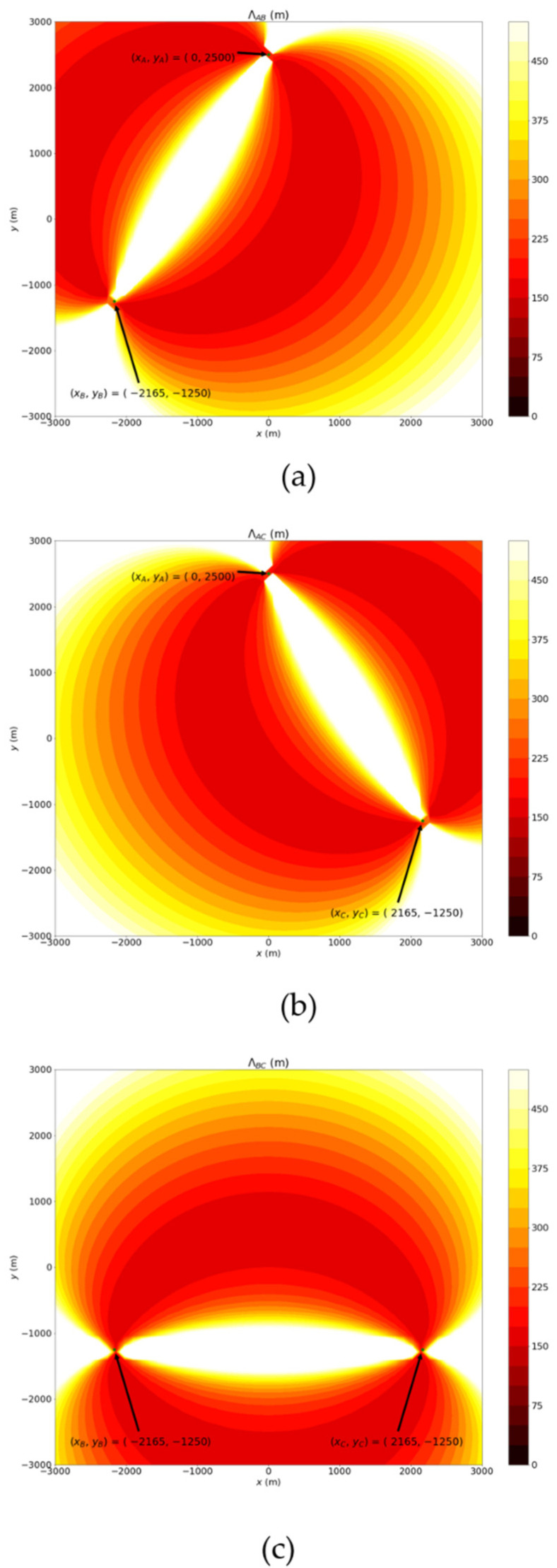
The example distributions of (**a**) *Λ_AB_* (**b**) *Λ_AC_* (**c**) *Λ_BC_*.

**Figure 6:**
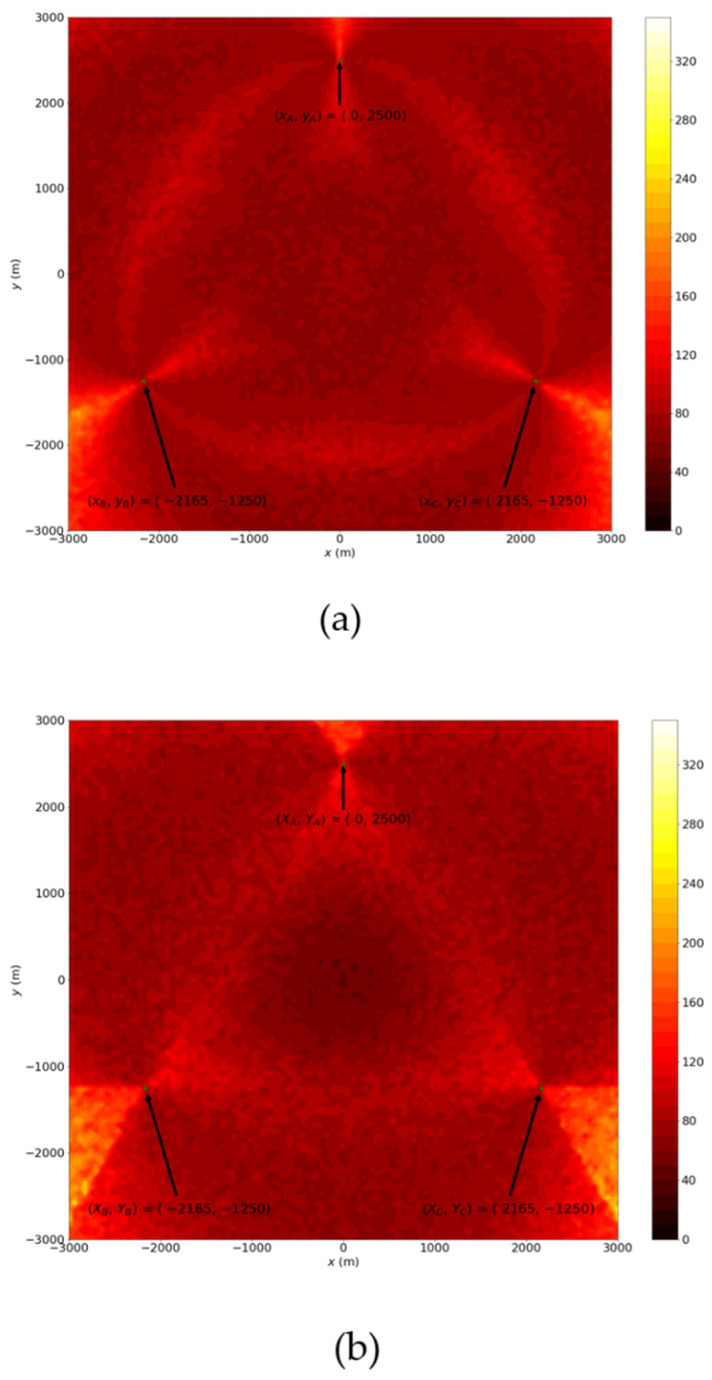
Simulated estimation error (**a**) without weighting; (**b**) with weighting.

First, the maximum distance error of these prediction points, $\Lambda_{AB}\left({\hat{P}}_{T,AB}\right)$, $\Lambda_{AC}\left({\hat{P}}_{T,AC}\right)$ and $\Lambda_{BC}\left({\hat{P}}_{T,BC}\right)$, can be obtained by Equations (3)–(15). Figure 5a–c show the distributions of $\Lambda_{i},\;i=AB,\;AC,\;BC$. Intuitively, if $\Lambda_{AB}\left({\hat{P}}_{T,AB}\right)$ is shorter than $\Lambda_{AC}\left({\hat{P}}_{T,AC}\right)$ and $\Lambda_{BC}\left({\hat{P}}_{T,BC}\right)$, the probability that the actual target located at the prediction result ${\hat{P}}_{T,AB}$ will higher than the probabilities of ${\hat{P}}_{T,AC}$ and ${\hat{P}}_{T,BC}$. However, suppose we chose the prediction result with the highest probability as the final estimation location of the target. In that case, the estimation error distribution will not be smooth, as shown in Figure 6a. Figure 6a shows the simulation result which selected the highest probability prediction when $\left(x_{A},\;y_{A}\right)=\left(0,\;2500\right),\left(x_{B},\;y_{B}\right)=\left(-1250\sqrt{3},-1250\right)$,$\left(x_{C},\;y_{C}\right)=\left(1250\sqrt{3},-1250\right)$ and $\;\Delta \phi_{A}=\;\Delta \phi_{B}=2{}^{\circ}$. Obviously, the error distribution inside the triangle region with three base stations as the vertices does not spread from the region center. The reason for this is that this method only considers one pair of stations at each point on the plane.

Hence, ${\hat{P}}_{T,AB}$, ${\hat{P}}_{T,AC}$ and ${\hat{P}}_{T,BC}$ should be considered together to make the error distribution smoother. Since each prediction result has its own probability, the final estimation result, ${\hat{P}}_{T,\;final}$*,* can be a weighted average location of the points ${\hat{P}}_{T,AB}$, ${\hat{P}}_{T,AC}$ and ${\hat{P}}_{T,BC}.$ Thus, the ${\hat{P}}_{T,\;final}$ can be computed by Equation (16):

(16) $${\hat{P}}_{T,\;final}=\frac{\sum_{i}W_{i}{\hat{P}}_{i}}{\sum_{i}W_{i}},\;i=AB,\;AC,\;BC,$$

where $W_{AB}$*,* $W_{AC.}$ and $W_{BC}$ are the weights of ${\hat{P}}_{T,AB}$, ${\hat{P}}_{T,AC}$ and ${\hat{P}}_{T,BC}$, respectively.

Moreover, the probability of the actual target being located in one of the prediction locations ${\hat{P}}_{T,i}$ is the reciprocal of the possible region’s surface area, ${\hat{P}}_{1,i}{\hat{P}}_{2,i}{\hat{P}}_{3,i}{\hat{P}}_{4,i}$ in Figure 2. To simplify the calculation complexity, we assumed that this area is proportional to the square of $\Lambda_{i}$, and then the probability is inversely proportional to $\Lambda_{i}^{2}$, as shown in Equations (17) and (18):

(17) $$A\left({\hat{P}}_{1,i}{\hat{P}}_{2,i}{\hat{P}}_{3,i}{\hat{P}}_{4,i}\right)\propto \Lambda_{i}^{2},\;i=AB,\;A.C.,\;BC$$

(18) $$P\left(target\;at\;{\hat{P}}_{T,i}\right)\propto \frac{1}{\Lambda_{i}^{2}},\;i=AB,\;A.C.,\;BC$$

where $\;A\left({\hat{P}}_{1,i}{\hat{P}}_{2,i}{\hat{P}}_{3,i}{\hat{P}}_{4,i}\right)$ denotes the surface area constructed by ${\hat{P}}_{1,i}{\hat{P}}_{2,i}{\hat{P}}_{3,i}{\hat{P}}_{4,i}$ and $P\left(target\;at\;{\hat{P}}_{T,i}\right)$ denotes the probability of the target at ${\hat{P}}_{T,i}$. Accordingly, the weight of the prediction location ${\hat{P}}_{T,i}$, i.e., $W_{i},$ can be set by $\frac{1}{\Lambda_{i}^{2}}$. Figure 6b shows the simulated error distribution of the estimation with the weighted method. Compared to Figure 6a, selecting the weighted estimation has a more balanced distribution than selecting the prediction with the highest probability.

## 4. Moving Target

To successfully track the moving target, the motion condition should be considered. When the angle estimation is varied and the stations are asynchronous, the error of determining target localization is increased. Hence, the efficient adaptive Kalman Filter is used to estimate the locations of a dynamic moving target via a series of incomplete and noisy measurements. The adaptive Kalman Filter algorithm is developed using the two-step procedure, including predicting and updating, to iteratively determine the locations of moving targets.

First, the state-space model is established to describe the tracking system by using state variables to obtain the moving target’s displacement, velocity and acceleration, as shown in Equations (19)–(29) [33,34]:

(19) $$x\left(n+1\right)=F\left(n+1,n\right)x\left(n\right)+v_{1}\;\left(n\right)$$

(20) $$y\left(n\right)=C\left(n\right)x\left(n\right)+v_{2}\left(n\right)$$

(21) $$Q_{1}\left(n\right)=E\left[v_{1}\left(n\right)v_{1}^{H}\left(n\right)\right],E\left[v_{1}\left(n\right)\right]=0$$

(22) $$Q_{2}\left(n\right)=E\left[v_{2}\left(n\right)v_{2}^{H}\left(n\right)\right],E\left[v_{2}\left(n\right)\right]=0$$

(23) $$Y_{n}=Span\left\{y\left(0\right),y\left(1\right),\ldots ,y\left(n\right)\right\}$$

where the state vector:

(24) $$x\left(n\right)=\left[\begin{array}{c} s_{x}\left(n\right)\\ v_{x}\left(n\right)\\ a_{x}\left(n\right)\\ s_{y}\left(n\right)\\ v_{y}\left(n\right)\\ a_{y}\left(n\right) \end{array}\right]$$

the transition matrix:

(25) $$F\left(n+1,\;n\right)=\left[\begin{array}{cc} \begin{array}{ccc} 1 & \Delta_{t}\left(n\right) & 0.5\Delta_{t}^{2}\left(n\right)\\ 0 & 1 & \Delta_{t}\left(n\right)\\ 0 & 0 & 1 \end{array} & \underline{\underline{0}}\\ \underline{\underline{0}} & \begin{array}{ccc} 1 & \Delta_{t}\left(n\right) & 0.5\Delta_{t}^{2}\left(n\right)\\ 0 & 1 & \Delta_{t}\left(n\right)\\ 0 & 0 & 1 \end{array} \end{array}\right]$$

the observation vector:

(26) $$y\left(n\right)=\left[\begin{array}{c} s_{x_{n}}\\ s_{y_{n}} \end{array}\right];$$

the observation matrix:

(27) $$C\left(n\right)=\left[\begin{array}{cc} \begin{array}{ccc} 1 & 0 & 0 \end{array} & \begin{array}{ccc} 0 & 0 & 0 \end{array}\\ \begin{array}{ccc} 0 & 0 & 0 \end{array} & \begin{array}{ccc} 1 & 0 & 0 \end{array} \end{array}\right]$$

the process noise vector:

(28) $$v_{1}\left(n\right)=\left[\begin{array}{c} n_{s_{x}}\\ n_{v_{x}}\\ n_{a_{x}}\\ n_{s_{y}}\\ n_{v_{y}}\\ n_{a_{y}} \end{array}\right]$$

and the measurement noise vector:

(29) $$v_{2}\left(n\right)=\left[\begin{array}{c} n_{s_{x_{n}}}\\ n_{s_{y_{n}}} \end{array}\right]$$

**Figure 7:**
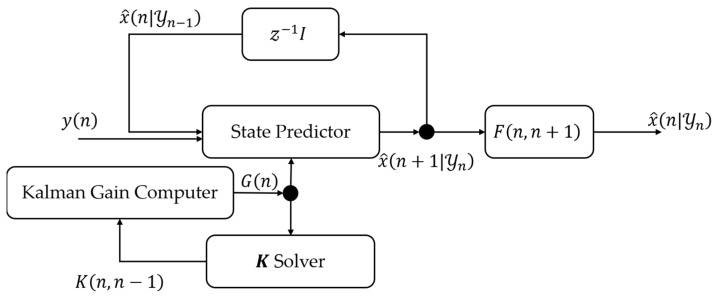
The block diagram of the Kalman Filter.

The block diagram of the Kalman Filter based on the state-space model mentioned above as depicted in Figure 7 and the recursive algorithm of the Kalman Filter is shown in Algorithm 1 [33]. First, before updating the state vector, the transition matrix should be calculated, as shown in Algorithm 2. Then, according to the Kalman Filter algorithm, we can update and memorize the state vector.
**Algorithm 1** Algorithm for Kalman Filter [33].Known parameters:1. $\hat{x}\left(n|Y_{n-1}\right)$: state vector predicted by $Y_{n-1}$ at  t=n−12. F(n+1, n): transition matrix at  t=n3. C(n): observation matrix at  t=n4. $Q_{1}\left(n\right)$: covariance matrix of system noise at  t=n5. $Q_{2}\left(n\right)$: covariance matrix of measurement noise at  t=nInitial conditions:6.  $\;\hat{x}\left(1|Y_{0}\right)=\left[\begin{array}{c} s_{x_{0}}\\ 0\\ 0\\ s_{y_{0}}\\ 0\\ 0 \end{array}\right]$ where $\;y\left[0\right]=\left[\begin{array}{c} s_{x_{0}}\\ s_{y_{0}} \end{array}\right]$
7. 
$K\left(0,-1\right)=\Pi_{0}$Input:8. y(n): prediction location of target at  t=n9. $\Delta_{t}\left(n\right)$: time difference between y(n) and y(n−1)
Output:10. Calculate F(n+1, n) by Algorithm 2.11. $G\left(n\right)\leftarrow F\left(n+1,\;n\right)K\left(n,\;n-1\right)C^{H}\left(n\right){\left[C\left(n\right)K\left(n,\;n-1\right)C^{H}\left(n\;\right)+Q_{2}\left(n\right)\right]}^{-1}$12. $\alpha \left(n\right)\leftarrow y\left(n\right)-C\left(n\right)\hat{x}\left(n|Y_{n-1}\right)$13. $\hat{x}\left(n+1|Y_{n\;}\right)=F\left(n+1,\;n\right)\hat{x}\left(n|Y_{n-1}\right)+G\left(n\right)\alpha \left(n\right)$14. $\hat{x}\left(n|Y_{n\;}\right)=F^{-1}\left(n+1,\;n\right)\hat{x}\left(n+1|Y_{n}\right)$15. $K\left(n\right)\leftarrow K\left(n,\;n-1\right)-F^{-1}\left(n+1,\;n\right)G\left(n\right)C\left(n\right)K\left(n,\;n-1\right)$16. $K\left(n+1,\;n\right)\leftarrow F\left(n+1,\;n\right)K\left(n\right)F^{H}\left(n+1,\;n\right)+Q_{1}\left(n\right)$
(Memorization/Delay)17.  $\hat{x}\left(n|Y_{n-1}\right)\leftarrow \hat{x}\left(n+1|Y_{n\;}\right)$18. K(n, n−1)←K(n+1, n)19. return $C\left(n\right)\hat{x}\left(n|Y_{n\;}\right)$


**Algorithm 2** Algorithm for Calculating F(n+1, n).Input:  1. $\Delta_{t}\left(n\right):\;$time difference between y(n) and y(n−1)
Output: 2. return $\left[\begin{array}{cc} \begin{array}{ccc} 1 & \Delta_{t}\left(n\right) & 0.5\Delta_{t}^{2}\left(n\right)\\ 0 & 1 & \Delta_{t}\left(n\right)\\ 0 & 0 & 1 \end{array} & \underline{\underline{0}}\\ \underline{\underline{0}} & \begin{array}{ccc} 1 & \Delta_{t}\left(n\right) & 0.5\Delta_{t}^{2}\left(n\right)\\ 0 & 1 & \Delta_{t}\left(n\right)\\ 0 & 0 & 1 \end{array} \end{array}\right]$


The memorized state vector will be utilized in the next update. Finally, the algorithm outputs the filtered prediction by multiplying the observation matrix  C(n) and the state vector $\;\hat{x}\left(n|Y_{n}\right)$.

As the algorithm shows in Algorithm 1, the $Q_{1}$ and $Q_{2}$ matrices should be defined before updating. According to the definition of $Q_{1}$ in Equation (21), we consider the $Q_{1}$ matrix as a tunable variable used for tuning the system performance. Moreover, the measurement noise matrix, $Q_{2}$, can be dynamically determined according to the predicted position of the target because it represents the possible measurement error of the predicted value. In our system, the measurement error of the estimated value $\left(s_{x},s_{y}\right)$ will vary with the relative position of the two base stations and the size of the quadrilateral enclosed by the two base stations, as shown in Figure 2.

**Figure 8:**
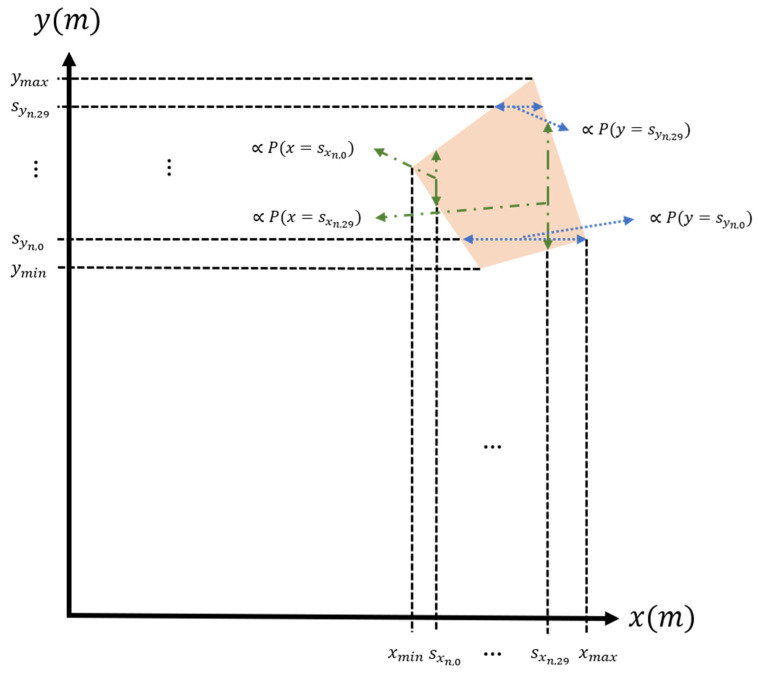
The example of calculating the probability $P\left(x=s_{x_{n,i}}\right)$ and $P\left(y=s_{y_{n,i}}\right)$.

According to Equations (22) and (29), the measurement noise matrix, $Q_{2}$, can be calculated by Equation (30). Before calculating the covariance matrix, the probability distribution along the x-direction and y-direction should be known first. Since the calculation time is limited, the probability distributions are determined by sampling 30 equal-spaced points corresponding to the x-direction and y-direction in the quadrilateral in Figure 2. Then we can get two sequences ${\left\{s_{x_{n,\;i}}\right\}}_{i\in \left[0,29\right]}\;$and ${\left\{s_{y_{n,\;i}}\right\}}_{i\in \left[0,29\right]}$. Since the probability distribution is uniform in this quadrilateral, the probability $P\left(x=s_{x_{n,\;i}}\right)$ and $P\left(y=s_{y_{n,\;i}}\right)$ is the proportion of the length in the y- and x-directions, as shown in Figure 8. Thus, the covariance of $\left(s_{x_{n}},\;s_{x_{n}}\right)$, $\left(s_{x_{n}},s_{y_{n}}\right)$ can be obtained by Equations (31) and (32).

In addition, the covariance of $\left(s_{y_{n}},s_{x_{n}}\right)$ and $\left(s_{y_{n}},\;s_{y_{n}}\right)$ are the same because they are real numbers, so they can be defined by Equation (33). Thus, we can update the $Q_{2}$ matrix by using Equations (30)–(33) before performing the Kalman Filter algorithm depicted in Algorithm 1, and then obtain the adaptive Kalman Filter algorithm.

Although the probability distribution of angle variation ±3° is assumed to be uniform, the probability$\;P\left(x=s_{x_{n,i}}\right)$ and $P\left(y=s_{y_{n,i}}\right)\;$still depends on the proportion of the length in the y- and x-directions in the quadrilateral, as shown in Figure 8. The other functions, such as Normal distribution and Gaussian distribution, can also accurately formulate the probability distribution of probable locations inside the quadrilateral. However, the complexity of the calculation will increase significantly because this distribution will extend to (−∞,∞). To accelerate the detection rate of passive DOA multiple sensor network, the x- and y-coordinates dependent probability distribution is adopted to achieve the efficient wireless localization system for moving targets.

(30) $$Q_{2}=\left[\begin{array}{cc} Cov\left(s_{x_{n}},\;s_{x_{n}}\right) & Cov\left(s_{x_{n}},s_{y_{n}}\right)\\ Cov\left(s_{y_{n}},s_{x_{n}}\right) & Cov\left(s_{y_{n}},\;s_{y_{n}}\right) \end{array}\right]$$

(31) $$\;Cov\left(s_{x_{n}},s_{x_{n}}\right)=\underset{i}{\sum }P\left(x=s_{x_{n,i}}\right)s_{x_{n,i}}^{2}-{\left(\underset{i}{\sum }P\left(x=s_{x_{n,i}}\right)s_{x_{n,i}}\right)}^{2}$$

(32) $$\;Cov\left(s_{y_{n}},s_{y_{n}}\right)=\underset{i}{\sum }P\left(y=s_{y_{n,i}}\right)s_{y_{n,i}}^{2}-{\left(\underset{i}{\sum }P\left(y=s_{y_{n,i}}\right)s_{y_{n,i}}\right)}^{2}$$

(33) $$\begin{array}{cc} Cov(s_{x_{n}},s_{y_{n}})= & Cov\left(s_{y_{n}},s_{x_{n}}\right)=\\ & \underset{i}{\sum }P\left(x=s_{x_{n,i}}\right)s_{x_{n,i}}P\left(y=s_{y_{n,i}}\right)s_{y_{n,i}}\\ - & \underset{i}{\sum }P\left(x=s_{x_{n,i}}\right)s_{x_{n,i}}P\left(y=s_{y_{n,i}}\right)s_{y_{n,i}} \end{array}$$

**Figure 9:**
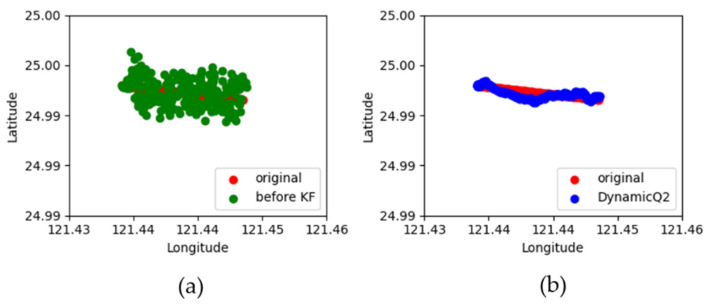
Simulation result of adaptive Kalman Filter. Comparison between the actual locations and the calculated locations by (**a**) using two-base station localization technique (**b**) combining two-base station localization technique and adaptive Kalman Filter.

**Figure 10:**
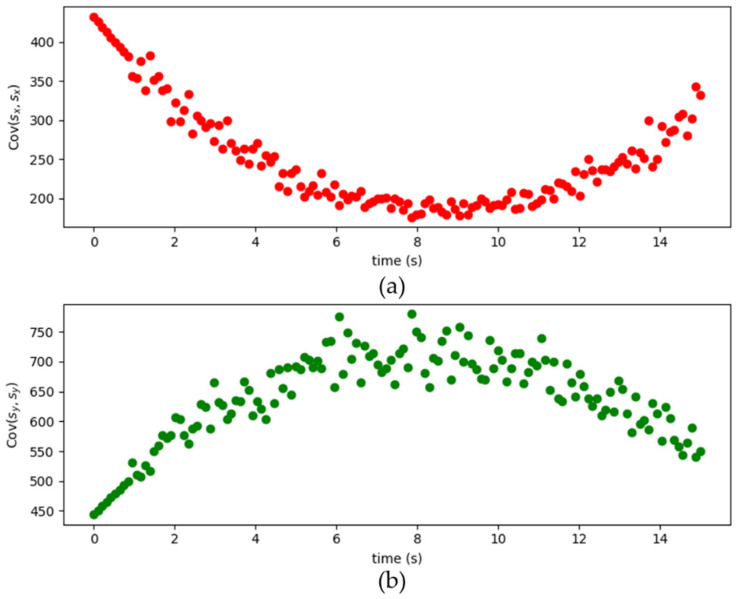
The (**a**) *Cov*(*s_x_*, *s_x_*) and (**b**) *Cov*(*s_y_*, *s_y_*) in the *Q*_2_ matrix.

Figure 9 shows the simulated results of moving target locations. The $Q_{1}$ matrix is $\left[\begin{array}{cc} 0.1 & 0\\ 0 & 0.1 \end{array}\right]$ and the dynamic matrices $Q_{2}$ of the measurement noise is shown in Figure 10. The $Cov\left(s_{x_{n}},s_{y_{n}}\right)$ and $Cov\left(s_{y_{n}},s_{x_{n}}\right)$ are both close to zero. Since the $Cov\left(s_{x_{n}},s_{y_{n}}\right)$ and $Cov\left(s_{y_{n}},s_{x_{n}}\right)$ are both close to zero, these figures only show the data of $Cov\left(s_{x_{n}},s_{x_{n}}\right)$ and $Cov\left(s_{y_{n}},s_{y_{n}}\right)$. In addition, the sampling time is set to 0.1 s. The “red dots” (original) are the actual locations of the moving target in a period of time (140 s). The “green dots” (before KF) are the original locations of the moving target by using the two-base station localization techniques presented in Section 3. The “blue dots” (DynamicQ2: Kalman with dynamic $Q_{2}$) are the calculated locations of the moving target by combining the two-base station localization technique and adaptive Kalman Filter discussed in Section 3 and Section 4, respectively. Results show that the blue dots agree well with the red dots, indicating that the location error can significantly decrease.

According to the simulation result, the location of target can be precisely predicted. Hence, the method we proposed can be extended to the 3D moving target detection by including the elevation angle determined by base stations. The longitude, latitude and altitude of the target can then be obtained by incorporating the GPS coordinates of the base stations.

## 5. Measurement

### 5.1. Single Base station

**Figure 11:**
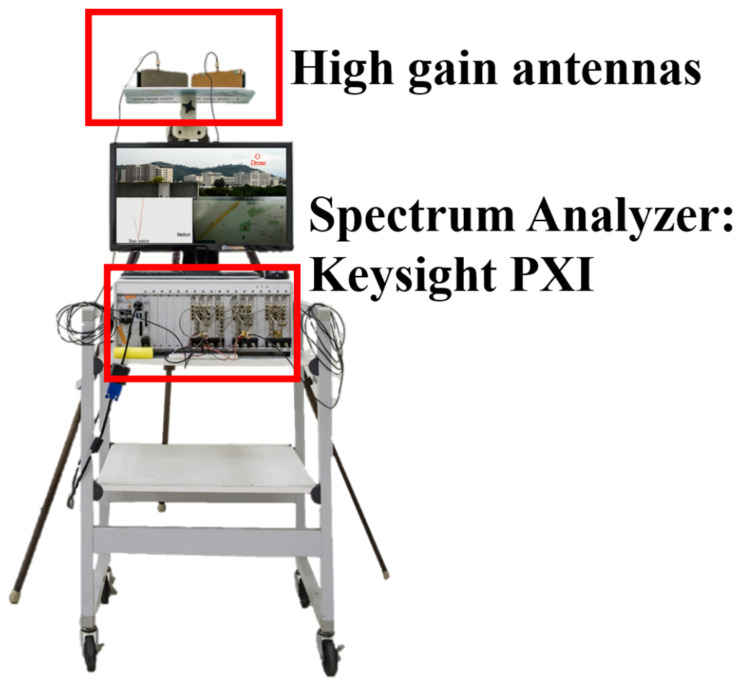
The antennas and spectrum analyzer used in the base station.

**Figure 12:**
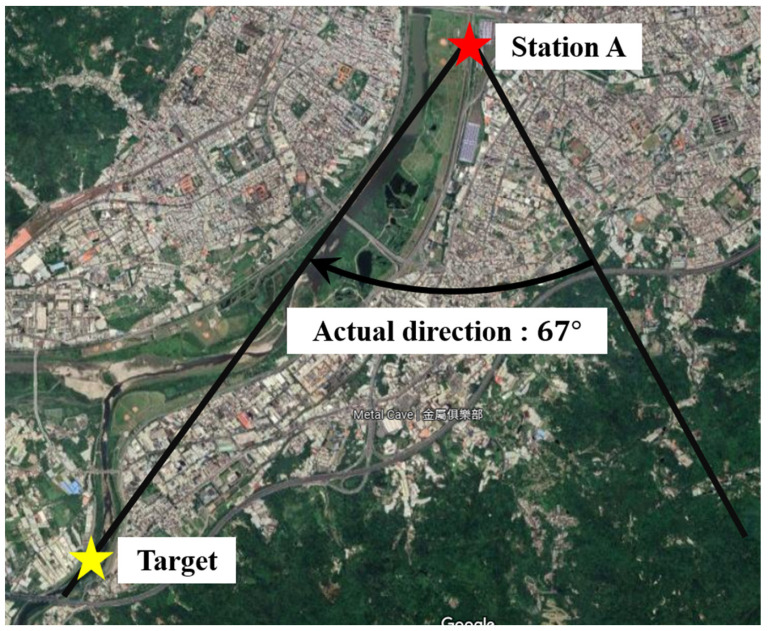
Single station measurement setup.

**Figure 13:**
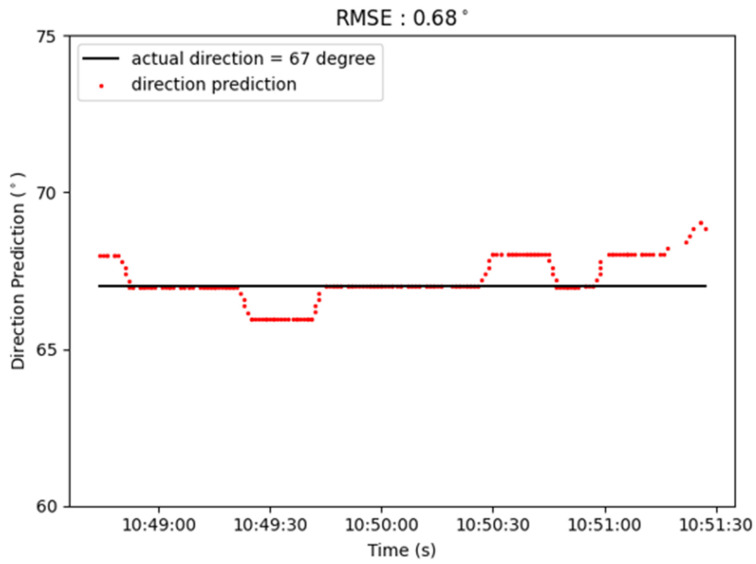
Single station measurement result.

This system adopts the conventional Direction of Arrival (DOA) technique as described in [29,30,31,32] to predict the target’s location. In our experiments, the base station in this system is constructed by the high-gain antennas and spectrum analyzer, Keysight PXI, as depicted in Figure 11. The detected signal frequency is about 5 GHz. In Figure 12, the distance between the single base station and the actual target is about 5.4 km. In addition, the azimuth angle of the target is 67 degrees. The positioning result of the fixed-point measurement during 90 s is depicted in Figure 13. The solid black line represents the actual angle of 67 degrees and the red dot represents the azimuth angle calculated by our proposed method. Its RMSE of location is about 0.68 degrees.

**Figure 14:**
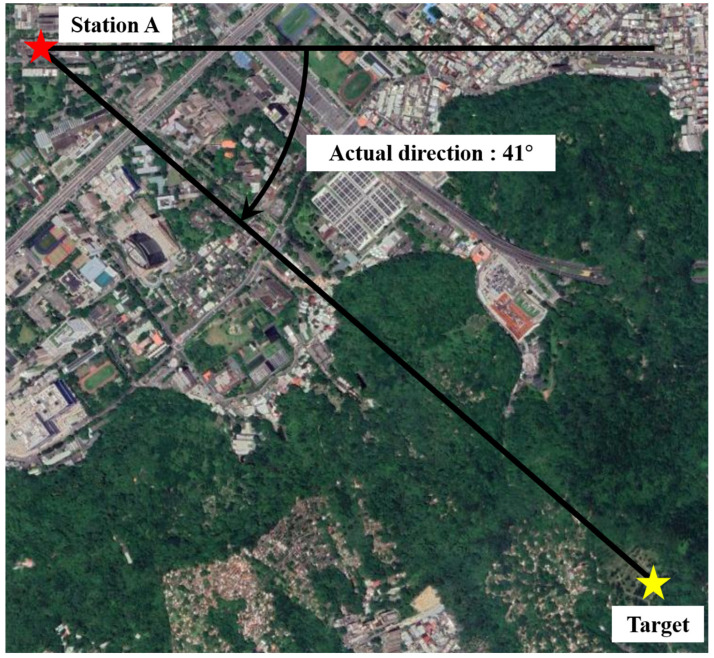
Single station measurement setup in the urban environment.

**Figure 15:**
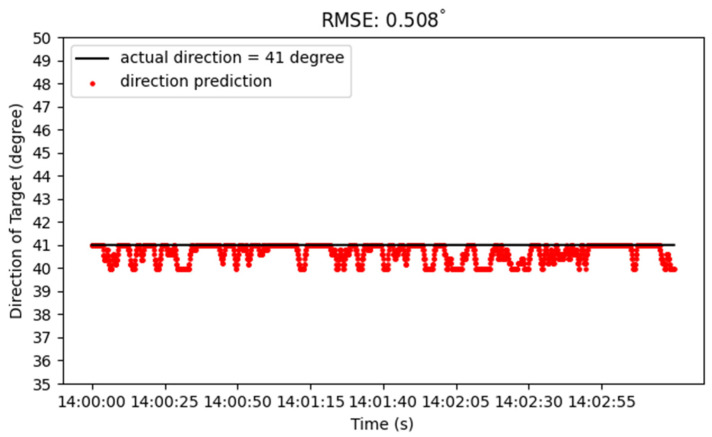
Single station measurement result in the urban environment.

Moreover, we further deployed this system in an urban region with rich interference signals in the environment. Figure 14 shows the experimental setup. The distance between the base station and target is about 1.9 km. In addition, the azimuth angle of the target is 41°. The positioning result of the fixed-point measurement during 180 s is shown in Figure 15. As presented in Figure 13, the solid black line represents the actual angle of 41° and the red dot represents the azimuth angle calculated by our proposed method. Its RMSE of location is about 0.508°. This small RMSE demonstrates that the system is reliable to predict the direction of target accurately even in a practical region with strong interference signals.

### 5.2. Two Basestations

**Figure 16:**
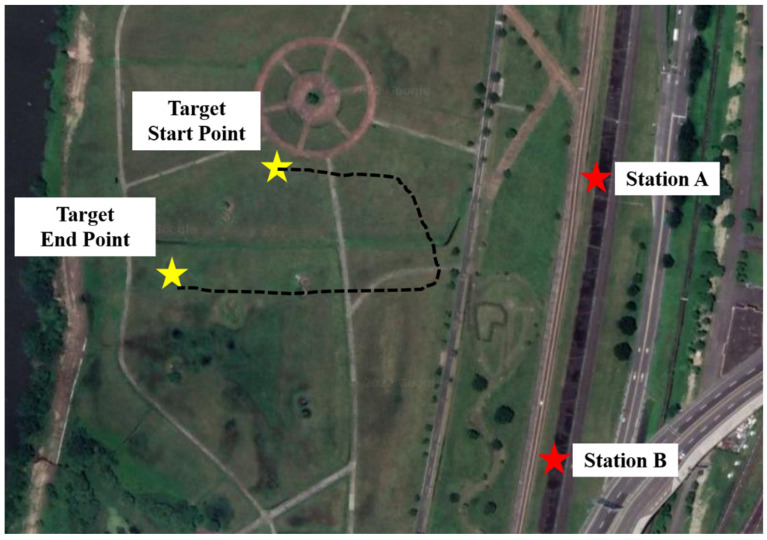
Two-base station system measurement setup.

**Figure 17:**
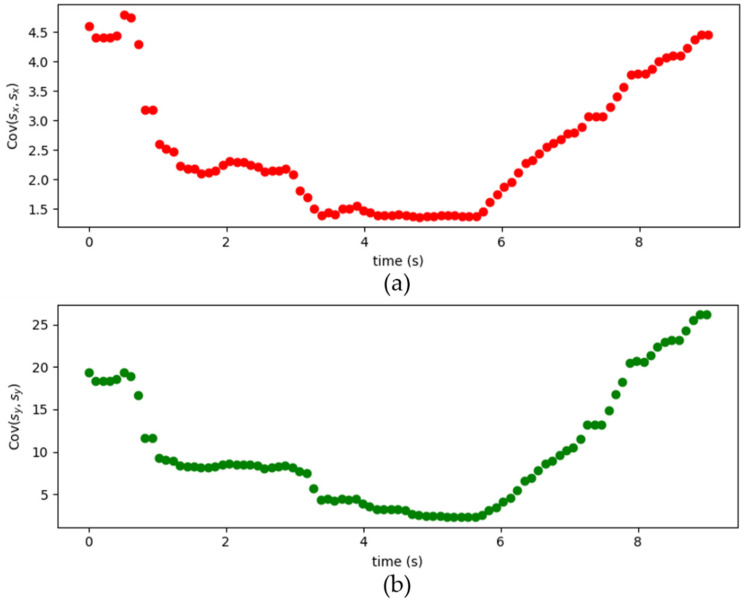
The (**a**) *Cov*(*s_x_*, *s_x_*) and (**b**) *Cov*(*s_y_*, *s_y_*) in the *Q*_2_ matrix.

**Figure 18:**
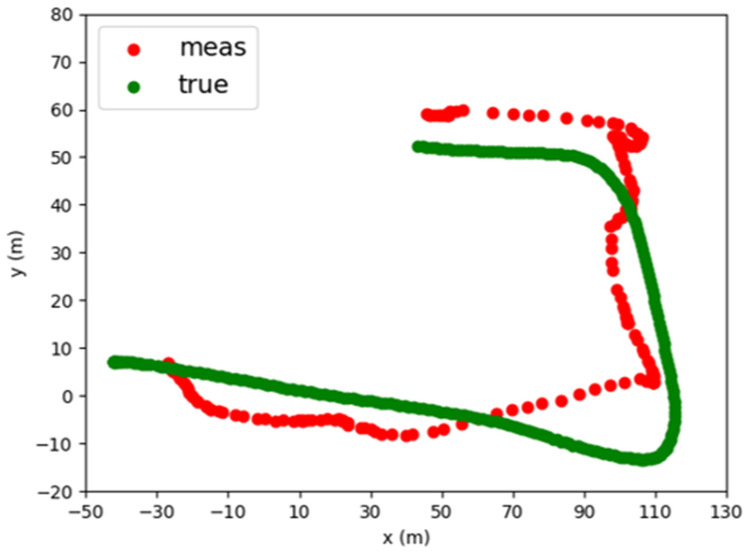
Two-base station system measurement result.

To demonstrate the presented wireless localization system using a DOA sensor network, the practical application scenarios in different target moving conditions are considered. This proposed DOA sensor network is designed to wirelessly detected moving target in $360^{{}^{\circ}}$ by using the operation of the adaptive Kalman Filter and hence the target with radiation signal can be effectively tracked and locked on even if its speed varies quickly and its trace is twisted and unstable. The setup positions of the two base stations and the actual moving target path are shown in Figure 16. The distance between the two base stations is about 168 m and the distance between the base station and the moving target is between 150 m and 300 m. The sampling time in the adaptive Kalman Filter is 0.2–0.5 s on average. The $Q_{1}$ matrix is $\left[\begin{array}{cc} 0.1 & 0\\ 0 & 0.1 \end{array}\right]$. $Cov\left(s_{x_{n}},s_{y_{n}}\right)$ and $Cov\left(s_{y_{n}},s_{x_{n}}\right)$ are shown in Figure 17 and the $Cov\left(s_{x_{n}},s_{y_{n}}\right)$ and $Cov\left(s_{y_{n}},s_{x_{n}}\right)$ in $Q_{2}$ matrix are both close to zero. The comparison between the calculated positioning result and the position of the moving target from the GPS receiver is shown in Figure 18. The green line is the real flight path of the moving target obtained from GPS and the red dot represents the calculated position of the moving target. The good agreement between the calculated data and the measured result for the single base station and the two-base station cases is observed, thus validating the usefulness of our proposed method.

## 6. Conclusions

The location estimation technique and the adaptive filtering algorithm of a passive localization system are proposed theoretically and experimentally in this paper. The single-base station and two-base station localization systems are implemented and the calculated and measured results of the fixed and moving targets are verified. By deploying this localization system at a $500\times 500{\;m}^{2}$ space, the average positioning error is only 10–20 m. The experimental results validate the effectiveness of the proposed multiple base station localization system in the practical application scenario. The 3-dimensional wireless localization system and the accelerated positioning algorithm is under investigated and will be presented in the near future.
